# Promoting safer gambling through social norms and goal setting: A qualitative process analysis of participants' experiences in the EROGamb 2.0 feasibility trial

**DOI:** 10.1016/j.invent.2024.100790

**Published:** 2024-11-20

**Authors:** Reece Bush-Evans, Emily Arden-Close, Sarah Thomas, John McAlaney, Ruijie Wang, Elvira Bolat, Sarah Hodge, Abigail Hamson-Ford, Keith Phalp

**Affiliations:** a Department of Psychology, Bournemouth University, Poole, United Kingdom; b Faculty of Health and Social Science, Bournemouth University, Poole, United Kingdom; c The Bournemouth University Business School, Poole, United Kingdom; d School of Sciences, Bath Spa University, Bath, United Kingdom; e Bournemouth University, Poole, United Kingdom

**Keywords:** Safer gambling, Social norms, Goal setting, Feasibility trial

## Abstract

Gambling, though a popular social activity, can lead to addiction and cause significant harm. This study aimed to explore the experiences of 36 low-to-moderate risk gamblers (PGSI score 0–7; 31 male, 5 female; 10 per each intervention arm, 6 per control group) in the ‘EROGamb 2.0’ feasibility trial (*n* = 168). The trial used social norm messages and goal setting feedback to promote safer gambling behaviour. Participants took part in semi-structured interviews via telephone or audio calls using Zoom or Wire, a secure messaging app. The interviews were analysed using Framework Analysis. Most participants found the interventions interesting and useful, though some reported no change in their gambling behaviour. Motivations for joining the trial included interest in the topic, altruism, and financial incentives. Participants appreciated the study's clear information, efficient processes, and helpful notifications, despite some technical issues. Reactions to social norm messages were mixed, with some expressing scepticism about the statistics. However, the goal setting intervention was well-received, with participants valuing the clarity and usefulness of the information. External factors, such as promotional offers from gambling companies, influenced gambling behaviour. The findings support the feasibility and acceptability of social norm and goal setting interventions to reduce gambling behaviour, highlighting the need for personalised approaches in future research.

## Highlights

• Social norm interventions are acceptable and feasible to deliver in reducing gambling behaviour.
• Goal setting interventions are acceptable and feasible to deliver in reducing gambling behaviour.
• Goal setting interventions may assist individuals in modifying their gambling behaviour.

## 1 Introduction

Gambling, often seen as socially acceptable, can escalate into addiction and pose serious problems for individuals and society (Gabellini et al., 2022; Wardle et al., 2019). In the UK, gambling's health-related economic burden is estimated between £1.05 to £1.77 billion annually (PHE, 2023). While those classified as experiencing ‘problematic gambling’ (PG), defined by a score of 8+ on the Problem Gambling Severity Index (PGSI; Ferris and Wynne, 2001), face significant health, economic, and social consequences; the broader population at lower risk of PG bears the greatest cumulative harm (Browne and Rockloff, 2018; Canale et al., 2016). This harm affects families, friends, workplaces, and communities (Azemi et al., 2023; Langham et al., 2015). Efforts are needed to prevent harm and provide tailored harm reduction strategies across the gambling risk spectrum (Latvala et al., 2019; Price et al., 2021; PHE, 2023).

A comprehensive public health strategy in the UK calls for customised prevention approaches to mitigate gambling risks and harms (Johnstone and Regan, 2020). However, recent reviews highlight the inadequacy of current interventions and their poor evidence base, underscoring the need for further research (Blank et al., 2021; Fiskaali et al., 2022; McMahon et al., 2019).

The gambling industry, recognised for its innovation, is closely linked to health-related risks (Schalkwyk et al., 2021; Thomas et al., 2023). With the rise of digital technologies and accessible internet services, gambling products have proliferated globally (Gainsbury et al., 2020; Glozah et al., 2023). Mobile devices, including smartphones and tablets, increase the risk of gambling-related harms (Gainsbury et al., 2016; James et al., 2016), as mobile users tend to place larger bets and gamble more frequently than computer users (Deans et al., 2016). Given the UK's high smartphone ownership (95 %; Ceci, 2019) and the success of text messaging interventions for low-to-moderate PG risk (So et al., 2020), digital harm reduction strategies need to be developed and tested (Lawn et al., 2020). The immediacy and accessibility of text messaging interventions may help users respond to urges or triggers in real-time, serving as preventative strategies for reducing gambling behaviours and gambling-related harm.

Research on brief electronic interventions for risky health behaviours, including alcohol use (Bewick et al., 2008) and problematic smartphone use (Kent et al., 2021), has been expanding (Giroux et al., 2017). Goal setting interventions, which involve setting and planning specific goals (Locke and Latham, 2019), have demonstrated robust effects on behaviour change across various contexts, including physical activity (McEwan et al., 2016) and smoking cessation (Lorencatto et al., 2015). Effective goal setting involves making goals public, setting them face-to-face with behavioural monitoring, and ensuring they are measurable and observable (Epton et al., 2017). Mobile devices offer opportunities for goal monitoring and feedback, aligning with goal setting theory (Eldredge et al., 2016; Locke and Latham, 2019).

Internet gambling facilitates rapid, uninterrupted play, which can exacerbate loss of control and loss chasing (Challet-Bouju et al., 2020; Mathieu et al., 2020). This makes goal setting interventions potentially useful for reducing gambling harms, helping individuals at moderate risk and PG adhere to expenditure limits (Rodda et al., 2017; Rodda et al., 2019; Rodda et al., 2022). However, single-session interventions may not be effective for non-problem or low-risk gamblers, as sustained behaviour change often requires repeat sessions (Michie et al., 2018). Since reducing gambling harm often involves multiple strategies (Rodda et al., 2022), it is crucial to investigate the acceptability and feasibility of goal setting interventions with end-users (Dowling et al., 2023).

Intervention, prevention, and harm reduction campaigns targeting social norms (rules and standards understood by group members that guide or constrain social behaviours; Cialdini and Trost, 1998) offer potential in reducing gambling-related harms (Marionneau et al., 2023; Miller and Prentice, 2016). Social norms include descriptive norms (perceptions of peer behaviours) and injunctive norms (perceptions of peer attitudes) (Dempsey et al., 2018). Social norms campaigns challenge misperceptions about peer behaviours and attitudes, often influenced by social psychological processes (McAlaney et al., 2011) such as attribution theory (Heider, 1958). Challenging these misperceptions encourages individuals to align with actual, less risky norms. Misperceptions have been observed in alcohol and substance use contexts among young adults (Arbour-Nicitopoulos et al., 2010; McAlaney et al., 2011), with social norms campaigns identified as cost-effective (National Institute on Alcohol Abuse and Alcoholism, 2015). However, such approaches are less studied in relation to gambling.

Past social norms campaigns typically used media (e.g., posters and advertisements), to reach the target population (Burchell et al., 2012; Schmidt et al., 2009), but exposure was minimal among target audiences (Bewick et al., 2021). Technology can enhance reach by generating personalised messages and delivering them directly (Dempsey et al., 2018). A recent meta-analysis recommended further exploration of social norms interventions to raise awareness of risky behaviours and reduce gambling harms, especially among lower risk individuals (Saxton et al., 2021). These interventions, due to their efficiency and low cost, could stimulate motivation and prompt contemplation of behavioural changes.

This qualitative study was nested within the 26-week, four arm, pragmatic, randomised controlled, EROGamb 2.0 feasibility trial (www.ISRCTN.org, registration 37874344; Arden-Close et al., 2023). The trial aimed to assess the feasibility and acceptability of online social norms and goal setting interventions to promote safer gambling in 168 low-to-moderate gamblers (PGSI score 0–7; Ferris and Wynne, 2001). Integrating qualitative research into trials enhances understanding of participant experiences and intervention acceptability (O'Cathain et al., 2015; Skivington et al., 2021; Yardley et al., 2015). In this study, participants provided feedback on their experiences of the interventions, trial participation, and processes. To our knowledge, no study has explored the feasibility and acceptability of interactive messages involving goal setting and social norms in online gambling contexts.

## 2 Method

### 2.1 Design

This study adopted a qualitative research design, comprising semi-structured interviews via telephone or audio calls using the secure messaging app Wire (used to deliver the feasibility trial interventions) or Zoom.

### 2.2 Trial arms

Participants in the feasibility trial were randomly assigned (1:1 ratio) to one of four research arms (goal setting, descriptive social norms, injunctive social norms, or control) for a six-week intervention period within the 26-week trial. Participants in the trial arms received weekly messages via Wire from the researcher, tailored to their assigned arm, prompting them to respond accordingly. In the social norms arms, participants received weekly text messages comparing their gambling behaviours (descriptive norms) or attitudes (injunctive norms) to those of others (see protocol paper for additional details; Arden-Close et al., 2023). In the goal setting arm, participants received guidance on setting goals through a short video and text message. They then set weekly goals for six weeks and were asked to self-report whether they had met those goals. Additionally, goal setting participants were requested to send screenshots (or a link, if applicable) of their objective gambling data each week, such as activity statements from the gambling operator via Wire. Participants in the control arm did not receive any intervention during the trial; but had the option to select their preferred intervention following trial completion.

### 2.3 Participants

Participants were recruited from the EROGamb 2.0 feasibility trial of social norms and goal setting for safer gambling, as detailed in the protocol paper (Arden-Close et al., 2023). Eligible participants were adults who gambled at least monthly and had a PGSI score of less than 8, indicating they were not at risk of problem gambling (Ferris and Wynne, 2001). Purposive sampling was used to ensure a diverse participant pool, considering demographic characteristics, baseline PGSI scores, and varying levels of engagement with the interventions (intervention arms only). Ten participants from each intervention arm and six from the control arm were interviewed. This recruitment strategy reached data saturation (Hennink and Kaiser, 2022), indicating that no new themes were emerging from the interviews.

Participants (goal setting: *n* = 10; descriptive social norms: *n* = 10; injunctive social norms: *n* = 10; control: *n* = 6) had a mean age of 56.31 years (SD = 13.38, range 24–72), were predominantly male (*n* = 31, 86.1 %), gambled daily (*n* = 23, 63.9 %), and identified as Caucasian (*n* = 32, 88.9 %). None reported having spoken to their GP about gambling. Twenty-five participants (69.4 %) reported betting more around sports events (e.g., prestigious horse racing events in the UK such as Cheltenham Festival and Royal Ascot). Thirteen (36.1 %) had used safer gambling tools (e.g., deposit limits, time-out), while 23 (63.9 %) had not. Devices used for betting included laptops (*n =* 25, 69.4 %), mobile phones (*n* = 32, 88.9 %), and iPads (*n* = 5, 13.9 %).

### 2.4 Procedure

The study was approved by the Bournemouth University Faculty of Science and Technology Ethics committee (ID: 33247). Interviews (*n* = 36) were undertaken between April 28th and September 28th 2022 by RBE (*n* = 26) a male interviewer, who delivered the intervention, and EAC (*n* = 10) a female interviewer, who designed the goal setting arm. Participation in the nested qualitative study was optional. The consent process for the feasibility trial included consent to be approached by researchers for a qualitative interview. Separate consent was obtained for the qualitative study. Both researchers had experience in conducting qualitative interviews. RBE contacted participants to confirm their willingness to participate, and interviews were scheduled at participants' convenience. Participants in the intervention arms were invited for interview after the 3-month follow-up, while those in the control arm were invited at the end of the trial.

Participants were informed that the qualitative study aimed to explore their experiences of the interventions, trial participation, and processes. A flexible topic guide, consisting of open-ended questions and follow-up prompts was developed by EAC, RBE, and ST. It covered trial participation (i.e., using Wire, completing questionnaires, payment), intervention experiences (i.e., experience of social norms messages, feedback received on goals, random assignment to a specific research arm), and gambling behaviour changes (changes made during/after interventions). The topic guide was reviewed by a Patient and Public Involvement (PPI) representative to help ensure it used inclusive and non-stigmatising language. Interviews ranged in duration from 45 to 60 min. Participants received a £20 Amazon voucher for their time. Interviews were audio-recorded and transcribed verbatim using Otter.ai, and transcripts checked for accuracy.

### 2.5 Data analysis

Transcripts were analysed by EAC using Framework Analysis (Gale et al., 2013). Field notes were taken immediately after the interviews. Following multiple readings for data familiarity and note taking, initial line-by-line coding was conducted on a subset of transcripts, cross-checked by AHF. A data-driven analytical framework was established, with codes grouped into categories. This framework was then used to create an Excel matrix for data charting. Iterative development of the analytical framework continued until saturation was reached. A summary of the analytical framework was shared with our two PPI representatives and their feedback informed the interpretation process. Following framework finalisation, data interpretation and report writing occurred.

## 3 Results

**Fig. 1:**
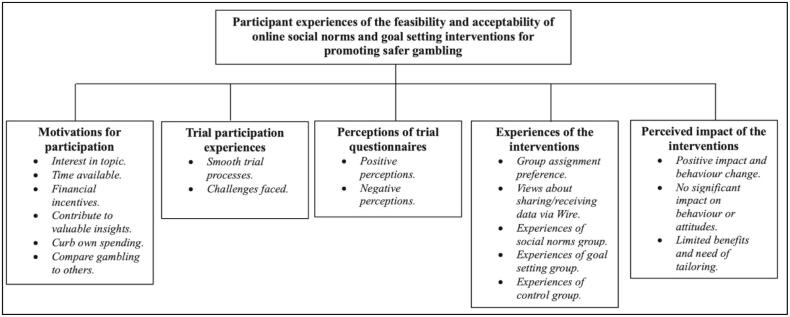
Themes and subthemes identified.

We identified five main themes (see Fig. 1) relating to motivations for participation (1), trial participation experience (2), perceptions of trial questionnaires (3), experiences of the intervention (4), and perceived impact of the intervention (5).

### 3.1 Theme 1: *Motivations for participation*

Most participants saw the trial advertisement on a gambling website or were referred by friends. Around two-thirds were motivated by a genuine **interest in the topic,** expressing curiosity about gambling behaviours:

*“I was interested in it really. I've always gambled. I've always not quite understood people who get out of control. And I just wanted to know more about it.”* [P067, injunctive].

Four participants mentioned having **time available** to take part in the trial, *“I suppose too much time on my hands.”* [P082, descriptive], while approximately half believed they could **contribute valuable insights** based on their gambling experiences:

*“I just thought there might not be that many people available of my kind of demographic. So, it might be quite useful for me to put my point of view in there” [*P036, control].

**Financial incentives** appealed to about one-third of participants, who appreciated the compensation offered: “*I liked the idea of the Amazon vouchers, to be honest”* [P066, injunctive].

Three participants were motivated by a desire to **curb their spending**:

“*I don't want to have the huge problems that [friend] got himself into. I gave up drinking for three months, because I was a bit worried about if I were an alcoholic. So, for the same reason, I welcomed the study. So, I should think more clearly about what I was doing* [P034, goal setting].

Finally, one participant **wanted to see how their gambling compared to others**:

*“I find it quite interesting to see how the study would actually capture people's gambling status to see if I am over gambling or normal.”* [P001, goal setting].

### 3.2 Theme 2: *Trial participation experience*

This theme comprised two sub-themes: smooth trial processes and challenges faced.

#### 3.2.1 Smooth trial processes

Overall, participants found the trial ran smoothly. They commended the **clarity and accessibility of information**, “*the amount was fine. It was pretty easy to read.”* [P058, descriptive]. **Notifications and reminders were appreciated** for keeping participants on track:

*“I'd get an email, please go to Wire and fill this out, I'd get a Wire notification. So, there was never a chance of slipping through the cracks”* [P055, goal setting].

Most found signing up, downloading, and using the Wire app straightforward: *“Simple, very, very easy. It's really just like downloading WhatsApp or another messenger*.” [P003, injunctive]. **Interactions with researchers were positive**, “*The method of interaction was good as well, very friendly”* [P069, descriptive], and participants found the **questionnaire layout and trial duration acceptable**: *“I think the length of study was just about right to capture relevant information.”* [P017, injunctive].

#### 3.2.2 Challenges faced

However, some participants encountered issues, such as difficulties with **downloading and using the Wire app and receiving Amazon vouchers**: *“I had to chase the first Amazon voucher.”* [P058, descriptive]. Some struggled with unfamiliarity with the Wire app and faced setup challenges:

“… *there was a slight problem with setting up Wire. That took a little bit of getting used to. To me, it would have been better to use WhatsApp.”* [P058, descriptive].

### 3.3 Theme 3: *Perceptions of trial questionnaires*

This theme comprised two sub-themes: positive perceptions and negative perceptions.

#### 3.3.1 Positive perceptions

Participants generally found the trial questionnaires **interesting**, *“It was interesting [to answer questions about others' gambling], because I didn't necessarily come across that information beforehand”* [P099, descriptive], prompting **reflection on gambling behaviours**: *“It's an eye opener for me. And I think it probably has contributed to me dumbing down and stemming my gambling as well, which is good.”* [P014, control]. Most felt **comfortable answering the questions** and **appreciated the inclusion of attention check questions in the questionnaires** to ensure data accuracy, “*I liked the trick questions in the poll, not trick, but just to make sure that people are answering properly.”* [P066, injunctive].

#### 3.3.2 Negative perceptions

However, one participant felt that the **language used in the questions presented an unbalanced view of gambling:**

“*I was irritated by them. because there wasn't a counterbalance to the assumption that gambling is harmful. I find gambling a very pejorative expression ... And it's got very negative connotation socially … I think the issue for me was that you're asking me to answer questions that were incredibly damage orientated. And if one actually sees  betting as a pastime, one realizes how important the use of correct language is, and how participants in the debate are influenced by language.”* [P040, descriptive].

Some participants found the questions repetitive or **difficult to answer accurately**, particularly those comparing their gambling to others:

“*There were questions in which I would have to guess how much people spent on a weekly basis on gambling, how many bets would they place? That sort of information was a pure guess, on my part.”* [P080, descriptive].

Additionally, some participants were **unsure why questions were asked about their physical health**, “*I wondered how gambling could affect physical mobility.”* [P026, descriptive], and others felt the **questions lacked options for nuanced response**: “*there was lots of questions about how often you feel or do something, and it would be ‘very often’, ‘several times this week’, ‘never’, I wanted the option ‘occasionally’.”* [P008, injunctive].

### 3.4 Theme 4: Experiences of the interventions

This theme comprised four sub-themes: group assignment preferences, views about receiving/sharing data via Wire, and experiences of the three interventions and control arm.

#### 3.4.1 Group assignment preference

Most participants (*n* = 21) expressed **no preference for group assignment**, *“I would have participated in whatever group I was put in.”* [P008, injunctive]. Some participants **preferred a specific intervention group** (goal setting (*n* = 4) or social norms (*n* = 7)), while a minority had no preference or expressed **a desire not to be in a particular group** (goal setting (*n* = 1) or control (*n* = 1)). Overall, participants accepted their allocation and were **satisfied with their assigned group**, “*As long as my input was valuable to the survey, I was happy with that,”* [P017, injunctive].

#### 3.4.2 Views about receiving/sharing data via wire

Nearly all participants were **willing to share their gambling data with researchers**, with one noting the possibility that **data sharing could itself lead to behaviour change**. Feedback on the **Wire messages was positive**, with participants finding that: “*the messages were very clear, and it was easy to follow”* [P020, goal setting].

#### 3.4.3 Experiences of the social norms intervention

Participants found the **social norms information interesting**, expressing intrigue in the comparisons with others: *“I think when you're compared to others, that's quite interesting.”* [P069, descriptive]. Some were **surprised by certain findings**, like the percentage of women who might hide gambling habits: “*I'm a bit surprised that 49%* [of women aged 35+] *sometimes might hide gambling”.* [P008, injunctive]. However, others found the **information aligned with their expectations**: “*You know, it didn't surprise me to be honest. Such a diverse field, any human sort of activity or whatever. Everyone is different.”* [P066, injunctive].

Some participants were **unsure of the basis of comparisons**: “*Well, you were comparing it to gender and age group, which, I suppose makes sense. But I don't know what those people are gambling on.”* [P094, descriptive]. They suggested **more specific comparisons by type of gambling** would have been helpful and proposed **obtaining information directly from betting companies** rather than via surveys.

#### 3.4.4 Experiences of the goal setting intervention

Participants generally found the **goal setting information and video helpful**: “*Good amounts of information very clear, easy to understand.”* [P103, goal setting]. Many were **familiar with SMART (specific, measurable, attainable, realistic, and time-bound) goals** and found them straightforward to implement: *“I've used smart goals before in my line of work … so I knew exactly what they were.”* [P103, goal setting].

#### 3.4.5 Setting goals

While some participants found **setting goals easy**, “*Because of my nature of gambling, which was basically based on promotions, it was very easy for me to set zero.”* [P020, goal setting], others **struggled to choose new goals**:

“*I must admit, at times I, I struggled to find the goals. But that sadly, that was more to do with having to find a different goal, as opposed to it may have been used to better bring another goal forward.”* [P045, goal setting].

Many participants reported that they **repeated their goals**: “*I just stuck to remembering what I did. Yeah, each time. Yes, stuck to the same. I think I adjusted a couple times, maybe 30 quid instead of 20”* [P077, goal setting]. Four participants thought the **goal planning was helpful**:

*“it's really helped. And first of all, I'm thinking, I can't do this. But once I use a SMART goal that you've given it was really easy.”* [P001, goal setting].

However, others **did not see its utility**: *“Maybe with goal setting, I would slash the planning bit”* [P020, goal setting].

#### 3.4.6 Achieving goals

Most participants reported **achieving their goals**, leading to a sense of accomplishment: “*I think it was more or less like 20 pounds for that period. And, yeah, I genuinely stuck to it”* [P077, goal setting]. However, some participants struggled to achieve their goals due to **unexpected offers or free bets**:

*“there was a few times where I think I'd set a goal that I wasn't gonna deposit anything that week. And then I got to Friday or Saturday and Sky had an offer…where it's events for something really simple to happen for MoSalah to have one shot and they boost the odds to even so if you place 10 pounds back and double your money…And then someone texts me and said, Sky got one of those offers on. So then I deposit my 10 pounds”* [EG055, goal setting].

This led to some participants **feeling disappointment**: *“slightly disappointed in myself. I think also from the fact that I was setting myself that I should have achieved it basically”* [P045, goal setting]. **Feedback on goal progress** varied, with some finding it encouraging: “*it was quite nice having the acknowledgement that my answers were being looked at. And then it just wasn't an automatic response.”* [P085, goal setting], and others feeling it was automated: “*like automatically generated things.”* [P034, goal setting].

#### 3.4.7 Accountability

Some participants **felt accountable to the researcher** when they were asked to report each week on their goal setting and attainment:

*“Knowing that you're monitoring me. I had to report on what I have done. I was thinking I mustn't go over. I had to tell you I hadn't met it [goal], and I'm like oh, great. I think it is because I had to report in on Wire. Having to say that I haven't met it or have gone over, it didn't make me feel great. And it made me realize I haven't kept to it”* [P001, goal setting]*.*

However, one participant mentioned some **technical issues with providing requested information**: “*… the information that you had to ask for wouldn't be totally ready until later on the Monday.* [P034, goal setting].

#### 3.4.8 Desire for additional support

Some participants expressed a need for **more support in setting goals**, particularly choosing appropriate goals and ensuring they were ‘SMART’:

*“I think anyone setting goals needs to have more help. When at the goal setting stage, choosing appropriate goals and making sure they're SMART targets, and just fine tuning and refining those goals ... it'd be very, very useful.”* [P103, goal setting].

They suggested discussions with researchers about goal setting could be beneficial: “*I think when I'm choosing a goal, it'd be helpful to take maybe 5, 10 minutes to discuss what will be a good goal”* [P103, goal setting].

#### 3.4.9 Utility of goal setting

Overall, participants viewed **goal setting as a useful strategy**, “*I liked the goal setting method because it helps you measure, and you can slow down the ramp and then adjust accordingly.”* [P020, goal setting], and beneficial in limiting their gambling expenditure:

“*it's a really useful tool to have. And it's really helped me so I'm pretty sure a lot of people who don't have that could help them. I mean, just being able to limit yourself is really useful*” [EG001, goal setting].

#### 3.4.10 Experiences of the control group

Participants in the control group accepted their assignment, **understanding the necessity of randomised designs** to evaluate interventions: “*Yeah, I think that's the appropriate way to do it [random assignment to groups]”* [P014, control]*.* Most were **patient with the waiting period** for the intervention, *“just fine, I waited; I thought you guys need to process all information”* [P018, control], although **some found it lengthy**: *“I think six months is quite a long time”* [P002, control]. Some participants were **curious about the activities of the other intervention groups**: “*I kind of thought I wonder how many messages they're doing?”* [P036, control].

### 3.5 Theme 5: *Perceived impact of intervention*

This theme comprised three sub-themes: positive impact and behaviour change, no significant impact on behaviour or attitudes, and limited benefit and need for tailoring.

#### 3.5.1 Positive impact and behaviour change

Some participants reported that they **gained insights into others' gambling habits**: “*It just gave me an idea of what other people's gambling habits were*.” [P099, descriptive]. This led them to reduce their betting, *“I think I've reduced on average the amount that I spend each week.”* [P045, goal setting], or reassess their own behaviour:

*“I think, do I get upset when I lose money? And then I think maybe, and I ask myself, do I chase losses? I think no, I don't actually, but it's good to have a reality check”* [P008, injunctive].

They felt the interventions had the potential to **help others recognise potential issues** with their gambling habits: “*It could indicate to those people that they may well have an issue.”* [P017, injunctive].

#### 3.5.2 No significant impact on behaviour or attitudes

However, some participants **did not contemplate or question their behaviour or attitudes** towards gambling, “*Well, there was nothing in the survey that would have caused me to have any reason to question my behaviour.”* [P040, descriptive], feeling their gambling was already controlled “*I consider my gambling to be controlled. And therefore, I'm not sure I fit into maybe somebody else's boxes.”* [P003, injunctive].

Some participants found the social norms information **interesting but not useful**, “*Useful? I don't think it was useful. It was interesting”* [P069, descriptive], and reported no change in their gambling behaviour: *“it didn't like change anything about what I was doing.”* [P008, injunctive]. This applied mainly to the social norm groups, some of whom felt goal setting might have been more effective in changing their behaviour.

#### 3.5.3 Limited benefit and need for tailoring

Participants recognised that the interventions **might not benefit everyone**, especially frequent gamblers lacking control: “*I'm not so sure for what I call hard gamblers. I don't know how they would be interested, really ... they don't seem to have any control”* [P077, goal setting]. They suggested that interventions need to **be tailored to different types of gambling**, accommodating the significant differences between sports betting and casino games:

*“there was no attempt made in the study, to differentiate between the millions of people who go to a race meeting and have a bet, as opposed to those who use casinos online, which, as far as I'm concerned, should be banned completely”* [P040, descriptive].

## 4 Discussion

In this nested qualitative study, we explored participants' experiences in a feasibility trial involving social norm messages and goal setting feedback aimed at promoting safer gambling behaviour. Overall, most participants had positive experiences, finding the interventions interesting and useful. However, some found the feasibility trial questionnaires repetitive or challenging, and a few reported no change in their gambling behaviour or attitudes.

Participants were primarily motivated to join the trial due to their interest in the topic, recommendations from friends, and a desire to contribute to research. This aligns with previous research suggesting altruistic motives (e.g., belief in research) may influence retention in longitudinal studies (Odierna and Bero, 2014; Price et al., 2016). Financial incentives and a desire to limit gambling spending also played a role, highlighting varied motivations among participants. While financial rewards are commonly cited as a primary motivator for participation in research trials (Almeida et al., 2007; Ferguson, 2008), our findings suggest that intrinsic curiosity and a desire for self-insights were also relevant factors.

Participants overwhelmingly reported positive experiences with the trial procedures, highlighting the clarity of trial information, efficiency of processes, and helpful notifications. Interactions with the researchers were also perceived positively. These findings underscore the significance of regular contact with research staff and direct communication with participants in research (NIH Clinical Center, 2016; Zweben et al., 2009). Some participants encountered technical issues when downloading and using the Wire app, emphasising the importance of testing app feasibility, usability, and acceptability before trial implementation, especially considering user characteristics and app design features (Birkhoff and Moriarty, 2020).

While most participants found the trial questionnaires engaging and thought-provoking, some struggled with questions regarding others' gambling behaviours and attitudes. People may not always consider social norms when assessing others' behaviours (Krupka and Weber, 2009), particularly if they do not participate in social gambling activities (many people gamble online alone). Additionally, the complex nature of online gambling, characterised by high accessibility and anonymity (Gainsbury et al., 2020; Papineau et al., 2017), adds further challenge to understanding others' perceptions and behaviours (Turner et al., 2007). This reflects one participant's point that there are important distinctions between different types of gambling, such as sports betting versus online casinos.

One participant expressed dissatisfaction with the language used in the questionnaires, highlighting concerns about certain terms being pejorative and potentially harmful. Terms like ‘problem gambling’ carry specific definitions but can inadvertently contribute to social stigma and personal distress (Blaszczynski et al., 2020). This underscores the importance of understanding the language used in research instruments, such as the PGSI (Ferris and Wynne, 2001) and its potential biases (Samuelsson et al., 2019). In designing interventions for the trial, considerations were made to avoid fear appeals or moralistic language. Both the social norms and goal setting interventions utilised neutral language and provided non-threatening feedback on participants' behaviours.

Participants' experiences within the intervention groups provided valuable insights into the feasibility and acceptability of aspects of the trial. Most participants did not have a strong preference for group allocation, aligning with the principles of randomised controlled trials (Schulz and Grimes, 2002). Additionally, participants demonstrated trust in the research process, as evidenced by their openness to share gambling data (i.e., links, screenshots). However, one participant noted that the process of sharing personal gambling data could have had an intervention effect and led to behaviour changes, highlighting an important consideration in the design of a possible future trial (Struminskaya et al., 2021).

Participants assigned to the control group were generally accepting of their allocation, acknowledging the importance of random assignment in trials. While most had no issues with the waiting period, a few expressed concerns about its duration, suggesting that a more dynamic communication approach could alleviate potential feelings of being left out or uninformed about the trial's progress. In a future trial, we would include a study newsletter, regular communications, and other behavioural science informed approaches to enhance engagement and retention (Gillies et al., 2021).

Participants in the social norms groups found the information interesting, consistent with social psychology research indicating a motivation to learn about peers' behaviour (Taylor et al., 2010). However, some expressed surprise or scepticism regarding certain social norms statistics. Stock et al. (2020) observed similar surprise reactions in adolescents regarding alcohol and drug use norms but noted greater acceptance than in our study. Differences may stem from societal attitudes towards gambling, potentially leading to defensiveness about gambling behaviours and attitudes compared to alcohol and drug use.

Previous research indicates that individuals who gamble tend to underestimate their losses, potentially affecting perceptions of the difference between their behaviours and those of others (Foster et al., 2012). Despite social norms messaging specifying the gender and age group for comparison, some participants were unaware who they were being compared to. This highlights the complex relationship between gambling and perceived norms. Additionally, social norms are often shaped by visible cues (Fay et al., 2012), which may be limited by the anonymity offered by online gambling (Fulton, 2019). Some participants suggested that more specific comparisons by type of gambling would have been helpful, emphasising the importance of personalised feedback tailored to individual demographics, behaviours, and attitudes. This point has been acknowledged within the social norms literature - though providing more granular, personalised social norms messages requires a larger baseline sample to identify misperceptions (McAlaney et al., 2011).

Participants provided positive feedback on the clarity and usefulness of the goal setting information, consistent with previous research (Rahimi-Ardabili et al., 2020). Setting and achieving goals was perceived as valuable, although some participants suggested that additional support might have been beneficial during goal selection and interaction with the researcher, aligning with recommendations for enhancing intervention effectiveness (Jeong et al., 2021; Locke and Latham, 2013). Despite most participants reporting goal achievement, in some instances goals were not met, often influenced by external factors like promotional offers from gambling companies. Such offers have been associated with increased gambling beyond limits in the general population (Hing et al., 2014). While randomised controlled trials minimise confounding factors (Spieth et al., 2016), practical limitations exist in controlling all risk factors for gambling harm, including exposure to gambling-related advertising and promotions (McGrane et al., 2023).

Participants reported varied intervention impacts. Some felt they had gained insights into others' gambling behaviours, prompting self-reflection and, in some cases, reductions in betting activities. This aligns with the recognised influence of social norms on behaviour (Cialdini and Goldstein, 2004; Kalkstein et al., 2023; Mennicke et al., 2018). However, others reported no changes in their gambling behaviours or attitudes, nor perceived control and saw no need for change. This may partly be attributable to the trial's eligibility criteria, which targeted participants with low-to-moderate PGSI scores (Ferris and Wynne, 2001). Given the individualistic framing of gambling responsibility (Marko et al., 2023), with many people considering themselves to be either in control and gambling ‘responsibly’ or having a ‘problem’ (Victorian Responsible Gambling Foundation, 2017), such responses were expected. Participants highlighted the need for interventions to be more tailored to address different types of gambling (i.e., sports betting, casino games). This emphasises the importance of personalised approaches to accommodate the diverse preferences and behaviours within the gambling landscape (Auer and Griffiths, 2022; Neighbors et al., 2015).

To our knowledge, this is the first qualitative study to explore the feasibility and acceptability of interactive messages involving goal setting and social norms in online gambling contexts. This innovative approach brings together behaviour change techniques that have been successfully applied in other areas, such as health interventions, but not fully explored in relation to gambling harm reduction. Given the increasing trend of online gambling (Tran et al., 2024), this study's insights into the acceptability of digital interventions could inform the development of wider public health strategies aimed at reducing gambling-related harm. However, the study has some limitations. Firstly, despite obtaining consent to access gambling data, practical challenges hindered data collection from multiple operators and assessment of land-based gambling expenditure was outside the trial's scope. As individuals who gamble online also tend to gamble offline (Kairouz et al., 2012; Leslie and McGrath, 2023), reliance on self-reported data may introduce biases, echoing challenges reported in other domains such as the use of brief electronic interventions for alcohol use (Bewick et al., 2021). Secondly, most participants were recruited from a single UK-based gambling operator, whose main market was sports betting. Replicating findings across a greater number of gambling operators encompassing a wider range of gambling activities would increase applicability of the findings. Lastly, the feasibility trial focused exclusively on those at low-to-moderate risk of gambling harm (PGSI score 0–7; Ferris and Wynne, 2001).

## 5 Conclusion

This qualitative study has provided insights into experiences of participation in the four-arm randomised controlled, EROGamb 2.0 feasibility trial of social norms and goal setting interventions to promote safer gambling behaviour (Arden-Close et al., 2023). All three interventions were feasible to deliver and deemed acceptable by participants. Experiences of the interventions varied, as did degree of contemplation and behaviour change reported. The trial design, including random assignment to intervention arms, was considered acceptable by the majority and trial processes were clear.

In a future definitive trial, it will be essential to include a wider range of gambling operators, ensure the trial design considers the implications of different types of gambling (e.g., sports vs. casino betting), provide regular communications (such as newsletters) to support retention (particularly for those assigned to a control group) and provide additional intervention support where needed. Overall, the findings highlight the importance of tailored approaches in interventions designed to support safer gambling and the need to accommodate diverse preferences and behaviours in trial design.

## Funding details

This work was funded by 10.13039/501100008625GambleAware. GambleAware is a grant-making charity using best-practice in commissioning, including needs assessment, service-planning, evaluation, and outcome-reporting to support effective, evidence-informed, quality-assured prevention of gambling harms. Guided by a public health model, GambleAware commissions integrated prevention services on a national scale and in partnership with expert organisations and agencies, including the UK National Health Service, across three areas of activity: universal promotion of a safer environment (primary); selective intervention for those who may be ‘at risk’ (secondary); and direct support for those directly affected by gambling disorder (tertiary). The authors alone are responsible for the views expressed in this article, which do not necessarily represent the views, decisions, or policies of the institutions with which they are affiliated. www.about.gambleaware.org.

## Declaration of competing interest

The authors declare the following financial interests/personal relationships which may be considered as potential competing interests: Bush-Evans reports financial support was provided by GambleAware. McAlaney is a member of the Gordon Moody Association Board of Trustees (unpaid role). If there are other authors, they declare that they have no known competing financial interests or personal relationships that could have appeared to influence the work reported in this paper.

## Data Availability

The data is not made available at this time due to the sensitive nature of the interviews and the risk of participant identification from the transcripts.
